# EEG theta and N400 responses to congruent versus incongruent brand logos

**DOI:** 10.1038/s41598-022-08363-1

**Published:** 2022-03-16

**Authors:** Hossein Dini, Aline Simonetti, Enrique Bigne, Luis Emilio Bruni

**Affiliations:** 1 Department of Marketing and Market Research, University of Valencia, 46022 Valencia, Spain; 2 The Augmented Cognition Lab, Aalborg University, 2450 Copenhagen, Denmark

**Keywords:** Decision, Perception

## Abstract

Neuroimaging and behavioral studies have shown that brands convey meaning to consumers. To investigate the immediate reactions of the brain to brand logos, followed either by congruent or incongruent pictorial brand-related cues, can deepen understanding of the semantic processing of brands, and perhaps how consolidated the logo is in consumers’ minds. Participants were exposed to different brand-related image sets, that were either congruent (a match between brand-related images and brand logo) or incongruent (a mismatch between brand-related images and brand logo) while having their brain signals recorded. Event-related potential and EEG time–frequency domain features were extracted from the signals of the target image (brand logo). The results showed significantly larger N400 peak and relative theta power increase for incongruent compared to congruent logos, which could be attributed to an error-monitoring process. Thus, we argue that brands are encoded deeply in consumers’ minds, and cognitive processing of mismatched (vs matched) brand logos is more difficult, leading to greater error monitoring. The results were mostly consistent with previous studies investigating semantic incongruences in the linguistic field. Therefore, the error-monitoring process could be extended beyond linguistic forms, for example to images and brands.

## Introduction

The world’s 100 most valuable brands reached a record value of 7.1 trillion U.S. dollars in 2021^1^. It is widely accepted that brands often represent the most important asset of a company and can influence purchasing decisions^2,3^. Neuroimaging and behavioral studies have shown that brands convey meaning to consumers^4,5^. However, how the brain connects brand elements (e.g., products) with brand representations (i.e., brand logo) is poorly understood. Thus, the immediate reaction of the brain to brand logos that are followed by congruent or incongruent pictorial brand cues can deepen our understanding of the semantic processing of brands.

Incongruence can be understood as a form of violation of pre-encoded rules or previous knowledge at the syntactic, semantic, or pragmatic levels, including contextual and background knowledge^6,7^. Because incongruences are most often unexpected, a violation of expectations may happen when they occur. Previous studies, mainly in the linguistic field, have found different brain responses to congruent and incongruent stimuli (see Baggio and Hagoort^8^ for a review of this topic). A specific electrophysiological marker related to congruence is the N400 event-related potential (ERP), a negative deflection in the electroencephalogram (EEG) signals that peaks around 400 ms after stimulus presentation. This marker was first found by Kutas and Hillyard, in 1980, who defined it as “an electrophysiological sign of the ‘reprocessing’ of semantically anomalous information”^9^ (p.1). After this initial work, several studies investigated the N400 effect on conflicting tasks (e.g., Stroop and flanker tasks)^10–15^, affective influences^16,17^, gesture representations^18,19^, sentences/words^20–26^, text and image^27,28^, and pictures^29^ (see Kutas and Federmeier for an extensive review of N400 studies^30^).

Brand logos are symbolic visual elements, consisting of image and/or text cues that aim to represent a brand in order to differentiate it from its competitors. They are so important that early definitions of “brand” could be summarized as “brand as a logo”^31^. In fact, competitive brands imitate features of leading brands, including brand logo, to benefit from brand equity of these leading brands^32^. Thus, it is crucial for companies that consumers associate a brand logo with the brand products and features. The N400 effect could indicate whether this link exists. Previous literature investigating semantic violations in sentence processing shows larger N400 amplitude, usually centro-parietally distributed, for words that are incongruent with a context, are infrequent, or have low cloze probability compared with congruent, frequent, or high cloze probability words^9,20–26^. However, studies using pictorial content as stimuli may be more relevant to this study as the brain could react differently to sentences as against images. Those studies provide evidence of the sensitivity of the N400 ERP to the semantic relationships between pictures^18,29^. Participants presented with pairs of either matched or mismatched pictures (e.g., knife-fork or cup-leaf respectively) had larger N400 amplitude (centering around 450 ms) broadly distributed over the scalp after a second mismatched picture, compared to a second matched picture^29^. Another study presented participants with words that were related or unrelated to succeeding pictures, regarding categorical or specific levels^33^. The N400 effect was found in the centro-parietal electrodes for all the manipulations, reflecting semantic mismatches in general. Gestural representations were investigated by presenting a short cartoon segment, followed by a short video with an actor reproducing the cartoon non-verbally (with spontaneous gestures)^18^. The video was either paired with the corresponding cartoon or with another cartoon segment. The results showed a wide, spatially distributed N400 effect, though more pronounced over the frontal and frontal-central midline sites, where a larger amplitude was found for incongruent than congruent gestures.

Overall, a violation of expectations seems to trigger the N400 response. It can be argued, however, that expectations exist because of previous knowledge of the world and of structures. It is therefore plausible to assume that memory is actively involved in stimulus processing. The findings of several studies suggest that the N400 effect reflects both the activation of working memory (e.g., immediate stimulus-context relationships) and also accessibility to long-term memory (e.g., context-independent relationships)^34^. Hence, stored knowledge related to a stimulus has to exist in the person’s mind in order to judge whether some piece of information is expected or not^18,34^. Indeed, the magnitude of the N400 effect is sensitive to the ease of retrieval of this previous knowledge, which can be interpreted as proportional to the cognitive load needed to process the stimuli^19^. Though such a time-domain EEG feature (i.e., N400) already indicates that memory plays a role in semantic processing—with implications for cognitive load—information from the EEG frequency-domain can confirm and extend the role of memory.

Neural oscillations pertain to the EEG frequency-domain analysis. The theta band—an oscillation in the frequency of 4–7 Hz—has been shown to differ in power depending on stimulus congruity level, where stimuli perceived as incongruent increase theta power compared to congruent stimuli^10–12,17,20,21,28,35,36^. Past studies suggest that the location of the theta activity indicates the type of process involved. For example, an increase in theta power over the posterior region (found for semantically incongruent words, though not for semantically congruent but unpredictable words) could simply reflect the detection of semantic incongruences^21^; whereas an increase in theta power over the midfrontal regions (found after presenting an incongruent word), possibly reflects an error-monitoring process^20^. Moreover, there is a relationship between theta power and memory^37^, including working and long-term memory^17,28^. In addition, the strength of the theta power is positively related to working memory demand^28,38^. The investigation of the semantic processing of emojis (pictorial representations of emotions or ideas) revealed that incongruent emojis—those emojis inconsistent with a sentential context—generated higher theta power at midfrontal, temporal, and occipital brain regions, compared with congruent emojis^28^. This was attributed to an increase in working memory load for error monitoring—represented by the midfrontal theta, and the activation of the long-term memory for emoji recognition and concept retrieval—represented by the occipital and temporal theta. However, theta increase in anterior parts (frontal) was also associated with retrieval of lexical information from long-term memory^17^.

Most of the aforementioned ERP and brain-oscillation studies of congruence effects focus on language (in verbal and non-verbal forms). Because brand logos can influence consumers’ brand perceptions^39^, investigation of the processing of brands by the brain can expand our understanding of how brands are represented in consumers’ minds. We therefore use real brands to explore how the brain reacts to brand-logos, representing brands that are congruently associated with brand cues (e.g., products, store layout), compared to logos that are incongruently associated with such cues. If brand logos are clearly represented in the minds of consumers, this knowledge should be accessible for retrieval when consumers encounter cues related to the brand. Thus, based on previous findings from other fields, we postulate that an increase in both N400 and theta power will occur in response to incongruent as against congruent logos. We propose that the N400 ERP and theta power features could be valuable for understanding how consolidated brands are encoded in the minds of consumers. Given our study design and stimulus, we expect to find a theta increase that represents an error-monitoring process, which is linked to working memory, as well as an activation of long-term memory. If this occurs, it could be argued that brand logos induce semantic processing that is similar to other representations, such as those encoded deeply in language.

## Results

This section describes the results of the ERP analysis focusing on the N400 component, followed by the results of the time–frequency (TF) analysis comparing congruent as against incongruent conditions in the theta band (4 to 7 Hz).

### Event-related potential

**Figure 1:**
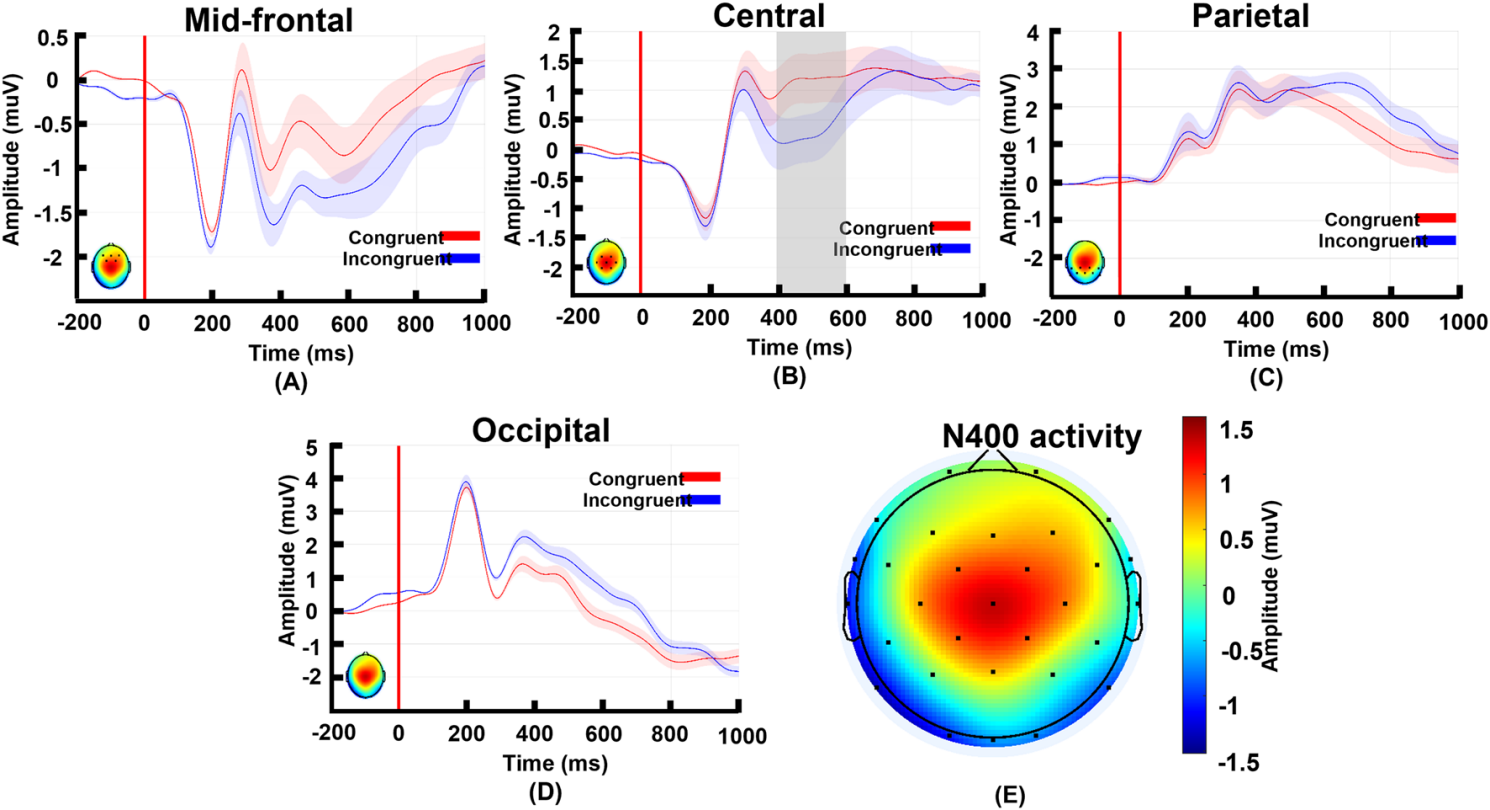
ERP obtained from average on trials of each condition and specific regions, and topo-map of N400 activity. Panels (**a**) to (**e**) show ERP activity for frontal, central, parietal, and occipital regions respectively. In all panels from (**a**) to (**d**), red curves show the ERP of congruent condition, and blue curve shows ERP of incongruent condition. The shaded area around each line indicates standard deviation of signals divided by square root of number of channels. In the same panels, the vertical red line in 0 ms indicates the start of the stimuli. The small topo-map at the bottom-left of each panel shows N400 activity and indicates the selected electrodes of each region. In panel (**c**), for the central region, the area highlighted in gray shows the significant difference between congruent and incongruent conditions (p = 0.04, effect size = 0.54), that occurs in N400. Panel (**e**) shows the difference of brain activity in two conditions (congruent-incongruent) in N400 (averaged from 400 to 600 ms). The hot colors show positive activity (i.e., congruent > incongruent) and the cold colors indicate negative activity (i.e., congruent < incongruent).

ERPs for each condition (congruent and incongruent) were obtained by averaging the corresponding pre-processed trials, in each specific brain region (mid-frontal, central, parietal, and occipital). Figure 1a–d shows the grand ERPs, which were obtained from the ERP average for all subjects in each condition. In the mid-frontal region (Fig. 1a), and the range of 400 to 600 ms, there is a pronounced difference between conditions, where the incongruent condition has greater negative activity than the congruent condition. However, a permutation test on the averaged data for the 400 to 600 ms time window revealed that this difference is not statistically significant (*p* = 0.19, effect size = 0.34). In the central region (Fig. 1b), there is a difference between the conditions in the range of the 400 to 600 ms time window, where the oscillations in the incongruent condition have greater negativity than in the congruent condition, which is statistically significant (*p* = 0.04, effect size = 0.54). In the parietal (Fig. 1c) and occipital regions (Fig. 1d), there was no significant difference in the 400 to 600 ms range between the two conditions (parietal: *p* = 0.84, effect size = − 0.05, and occipital: *p* = 0.27, effect size = − 0.29). Finally, Fig. 1e shows the averaged ERP amplitude differences (congruent minus incongruent) of each electrode in the 400 to 600 ms time window. The main difference occurred in the central region, which is consistent with a significant difference between the conditions occurring only in the central region.

### Time–frequency

**Figure 2:**
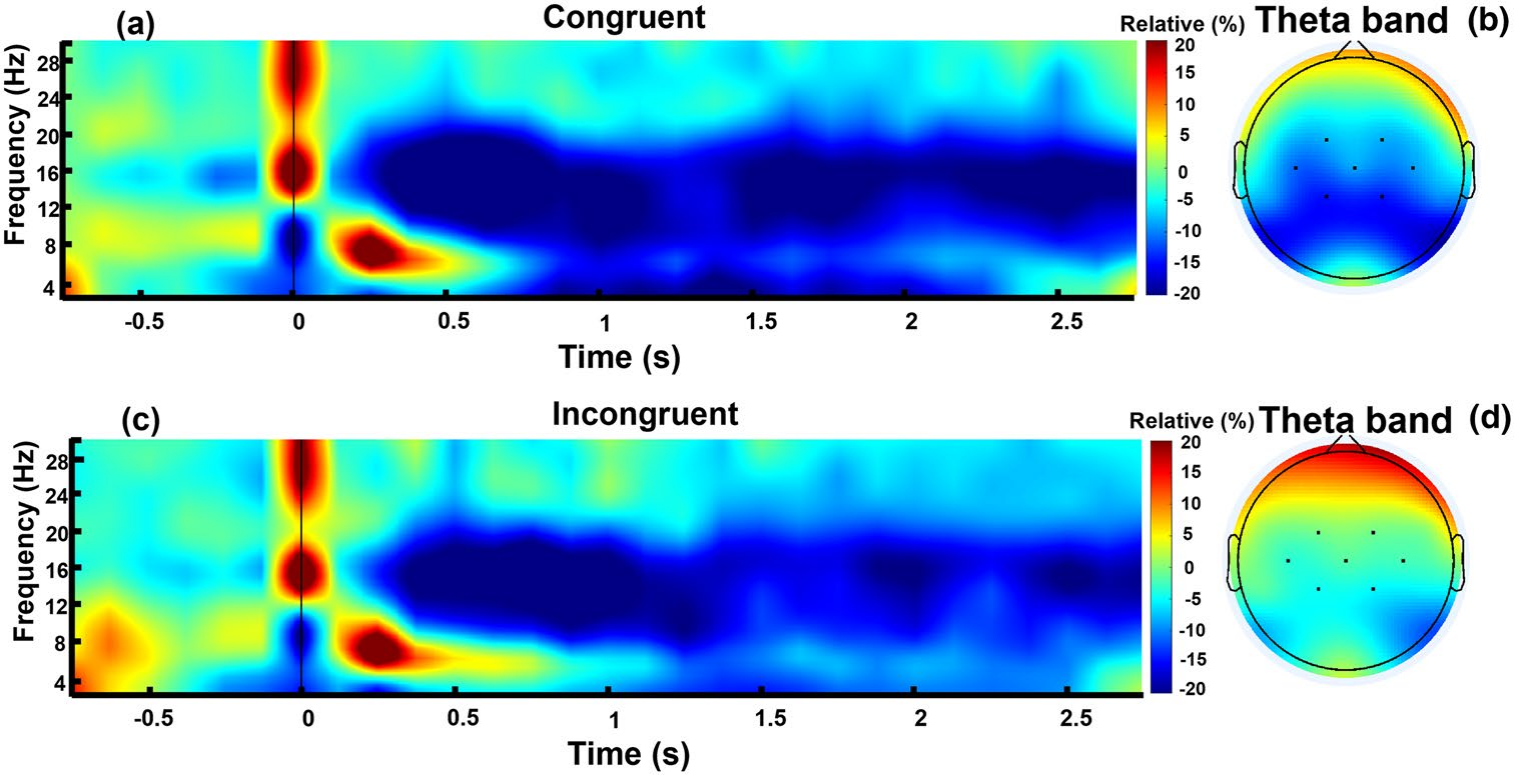
Time–frequency and topo-map (of theta band in central region) for congruent and incongruent conditions, calculated from brain activity of central region. Hot colors show positive relative power and cold colors show negative relative power. Panels (**a**) and (**c**) show time–frequency of congruent and incongruent conditions respectively. Both panels are plotted from − 0.75 to 2.75 s. The y-axis indicates frequency and the x-axis indicates time. The vertical black line in 0 s indicates the start of the stimuli. Panels (**b**) and (**d**) show topo-maps of brain activity in theta band of congruent and incongruent conditions respectively. The activity is averaged over theta (4 to 7 Hz) and time (0 to 2 s), and plotted as topo-maps.

Figure 2a,c show the TF activity of congruent and incongruent conditions in the central region respectively. As shown, in the theta band frequency, and from 700 to 2300 ms, there is a negative relative power in both conditions, where the congruent condition is more negative. In addition, there is a negative relative power in alpha and beta frequencies for both conditions, starting from around 200 ms and lasting until the end of the stimulus. Figure 2b,d show the scalp power spectrum activity for the congruent and incongruent conditions respectively. There is negative relative power activity in the central region in both conditions, but the strength of this negativity is higher in the congruent condition. Moreover, there is positive relative power activity in the mid-frontal region only in the incongruent condition.

**Figure 3:**
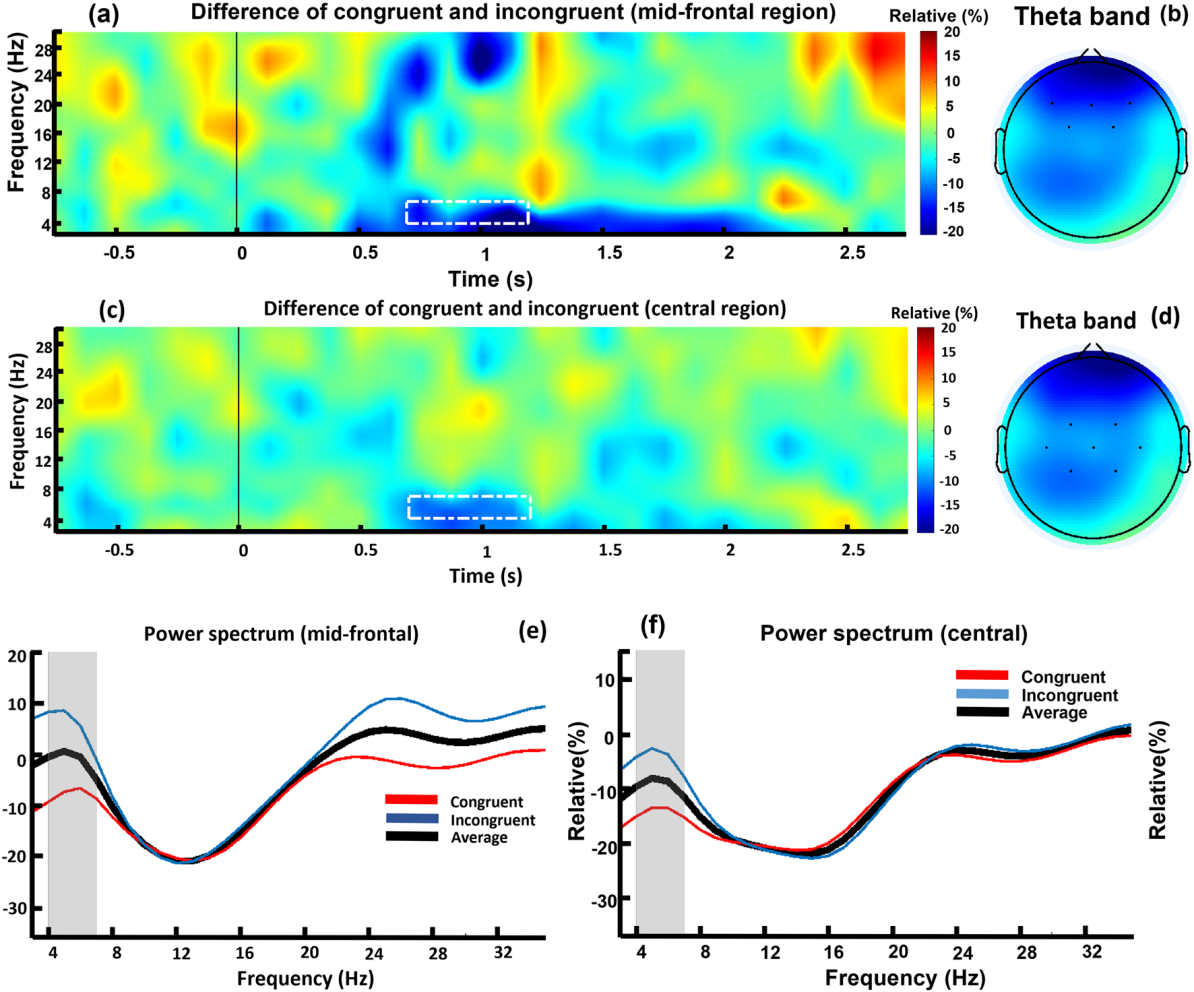
Time–frequency differences (congruent-incongruent), topo-maps (of theta band), and power spectrums. Hot colors show positive relative power and cold colors show negative relative power. Panels (**a**) and (**c**) show the subtracted TF of incongruent condition from congruent condition (congruent-incongruent). The white rectangle shows the region of theta frequency (4 to 7 Hz) and time (700 to 1200 ms), where the difference is maximum. The statistics, topo-maps, and power spectrum of this figure are all focused on this region. Panel (**a**) shows the TF difference in mid-frontal region and panel (**c**) shows the TF difference in central region. Panels (**b**) and (**d**) show topo-maps for mid-frontal and central regions respectively. They show brain activity averaged on the theta frequency and 700 to 1200 ms (the area of white rectangular). Panels (**e**) and (**f**) show the power spectrum activity of mid-frontal and central regions respectively. They show power spectrum of all frequencies averaged over time. The gray regions in both panels indicate the theta band, and there is significant difference between congruent and incongruent conditions both in mid-frontal (p = 0.03, effect size = − 0.54) and central (p = 0.01, effect size = − 0.69) regions. In both panels, the yellow curve shows the power spectrum of congruent, the red curve shows the power spectrum of incongruent, and the black curve shows the average power spectrum of congruent and incongruent conditions.

Figure 3a,c show the TF differences between the conditions (congruent minus incongruent) in the mid-frontal and central regions respectively. There is negative relative power in the theta band, and the 700 to 1200 ms time window (demarcated by the white rectangle), which occurs in both regions. We tested the statistical significance using a permutation test. The results showed that, in the mid-frontal region, activity is significantly higher for the incongruent condition compared with the congruent condition (*p* = 0.03, effect size = − 0.54), as well as in the central region (*p* = 0.01, effect size = − 0.69). Figure 3b,d show the differences in activity of the conditions in the mid-frontal and central regions respectively, obtained from averaging the same abovementioned frequencies and periods. The difference between conditions in the central and mid-frontal regions is noticeable. Finally, Fig. 3e,f show the power spectrum density of the mid-frontal and central regions respectively, which is the TF averaged over time. A significant difference occurs in the theta band, and it is specified by the gray area in the figures, where theta activity in the incongruent condition (blue line) is significantly higher than in the congruent condition (red line). Statistical analysis showed no significant difference between the two conditions in any other specified regions.

## Discussion

In this study, we evaluated the ongoing, neuronal, semantic processing of brand logos, using ERP and Welch-based relative power analysis. Participants were exposed to 80 image sets, where the last image of each set included a brand logo preceded by a set of three images including brand-related cues (e.g., products or services). The last image (i.e., the logo) could either be congruent or incongruent with the previous cues. We aimed to identify both time (ERP) and frequency (EEG power) changes that were anticipated as emerging because of semantic violation between brand cues and logos (hereafter, brand cues-logo). We therefore focused on N400 ERP activity and theta-band (4 to 7 Hz) power. In time-domain analysis, the results showed significantly larger negative N400 amplitude in the central electrode locations when incongruent logos were presented to participants, compared with congruent logos. In the frequency domain, incongruent logos led to significantly higher relative theta activity in the mid-frontal and central electrode locations compared with congruent logos, which can be further related to cognitive demands. The results suggest a neural distinction for semantic processing, between the congruent and incongruent semantic processing of brand logos. In the following paragraphs, we will first discuss the ERP and then the time frequency results.

Regarding the ERP, we found a significantly higher N400 peak in the central electrode locations for semantically incongruent brand cues-logo representations than semantically congruent ones. It is widely accepted that violations in semantic expectations result in larger higher N400 peaks in a wide variety of fields and tasks^9,25,29,40^. A review paper in the linguistic field suggested that the N400 reflects two main brain processes, unification and pre-activation, which are related to meaning integration. The authors declared that this activation is widely spread across brain electrode locations, but mostly focused in the frontal and temporal cortexes^8^. Studies using pictures as stimuli, which relate better to the present study, have also found a higher N400 for the incongruent than congruent condition. For example, Hamm et al. investigated brain responses to semantically congruent and incongruent images of different objects during an object-identification procedure^33^. Their results showed a greater N400 effect in the central-parietal electrode locations, and they concluded that N400 is responsible for semantic mismatch processing. In another study, Wu et al. showed a video-clip of a cartoon followed by an image of either a congruent or incongruent gesture^18^. They reported that the N400 is greater for the incongruent condition in the frontal electrodes, and claimed this is connected to semantic processing of gesture images. Moreover, many other image-based studies using picture series, line drawings, and videos as stimuli, reported greater N400 peaks in the frontal electrode locations^29,41–43^. Our results prove that, in line with previous studies, the N400 peak reflects the semantic processing of brand cues-logo associations. We argue that incongruence between brand logos and other brand cues represents a semantic violation that requires greater mental processing effort, to adjust to the violation, which is reflected by a high N400 peak. Although most previous image-based studies have reported frontal N400 (except Wu, Y. C. and Coulson, S.^18^, that reported the effect in the central-frontal electrode locations), our results showed the N400 effect in the central electrode locations. This could be because of our type of stimulus (i.e., brand logos), whereas most other studies used ordinary objects, gestures, or images, shown within a context of sentences. Contrary to most forms of visual representation, such as those used in previous studies (mostly iconic), brand logos are not so open to interpretation due to their symbolic nature (note that we are not referring to the creative elements of a logo, instead to what they intrinsically represent). They provide a direct, unique, and unambiguous connection with a particular brand, while most other visual elements can have multiple associations. It is therefore reasonable to expect differences in the N400 effect compared with other types of stimuli. Finally, it is worth mentioning that studies including conflict tasks (such as the Stroop or flanker tasks) have consistently found greater N400 in the frontal-central electrode locations for incongruent as against congruent conditions^10,12–15^. Even though they emphasize more the response in the frontal than the central electrode locations, these studies provide evidence of the role of the central electrode locations in tasks concerning conflict (incongruence). The present study does not have a conflict-oriented task, therefore, the role of the central electrode locations in semantic violation processing needs further investigation.

Regarding the time frequency analysis, consistent with previous studies showing a theta power increase in the frontal/mid-frontal areas with higher cognitive processing, incongruence between brand cues and brand logo representations in the present study led to an increase in the theta band. Previous studies, which mainly focused on linguistic information, found that the theta band is connected to improved cognitive processing of language-related tasks^20,44–46^, working memory demand^28,38^, long-term memory^17,28^, detection of semantic incongruences^21^, increased task difficulty, and higher attention demands^47,48^. In a study of the pictorial aspects of cognitive processing, Tang et al. declared that cognitive processing of “paralanguage information”, which is a category differentiated from linguistic processing, is also connected to theta oscillations^11^. Despite the fact that brand-related stimuli are neither linguistic nor emoji-based, the present findings showed that their cognitive processing is connected to theta oscillation. This suggests that theta oscillation indicates the neural activities occurring behind the detection of semantic violations.

In this study, we mitigated the influence of brand and picture (design differences) on brain responses by subtracting the responses to incongruent as against congruent brand logos. Semantic violations in incongruent brand cues-logo led to an increase in theta power in the mid-frontal electrode locations. Previous literature has declared that a theta power increase in the frontal and mid-frontal electrode locations is associated with difficulties in meaning integration, such as lexicon context and higher processing effort^28^. Moreover, mid-frontal theta increase possibly reflects an error-monitoring process^20^, and theta power increase could indicate higher working memory load in error monitoring^28^. Considering our current results concerning theta power increase, and previous studies, we argue that the processing of violations between brand cues and brand logos needs greater effort in integrating mismatched brand representations (i.e., logos) with previous knowledge about the brand. Consequently, there is high working memory load in monitoring the manifested error during prediction, and this cognitive load is reflected in higher theta activity in the mid-frontal electrode locations.

The findings also showed a significant theta power increase in the central electrode locations caused by semantic violation of incongruent brand cues-logo. To the best of our knowledge, there are not many studies investigating theta power responses to semantically incongruent image stimuli. Past results are mainly related to flanker and Stroop tasks, concluding that theta power increase is caused by conflict-related processing. Fernández et al. investigated theta activity in incongruent-vs-congruent trials in a Stroop task followed by a speech task^49^. They reported that the conflict caused by both tasks (especially the speech task), induced a theta power increase in the mid-central electrode locations. Using a flanker-type task, Pan et al. reported an increase of theta power for positive targets after incongruent rather than congruent primes in the central electrode locations^50^. They suggested that this theta increase was due to the integration of positive emotions with conflict resolution. Using an emotional conflict task, Ma et al. concluded that greater central theta activity in the incongruent (as against congruent) condition was due to a greater need for control in conflicting conditions^51^. Our results showed a significant increase in central theta. Because our task does not contain conflicting situations, it seems that this central theta increase reflects other kinds of processes. We found no previous EEG studies investigating brand logos or using related image stimuli. Therefore, further studies need to be done to have a better understanding of central theta increases in response to incongruent brand cues-logo.

Our data did not show a significant theta increase with incongruent processing, either in the occipital or the temporal electrode locations. Regarding the occipital electrode locations, a linguistic study stated that left-occipital theta power increase might be associated with visual form processing where longer and more complex words showed higher theta than shorter and simpler words^46^. Another study reported a theta power increase in the occipital lobe with emoji-processing compared to word-processing, which is possibly due to the complexity of the visual forms of emojis, which can be vague and difficult to retrieve^28^. One reason for dissimilar results might be because of our stimulus type. The abovementioned studies compared either two linguistic related stimuli together or a word/sentence stimulus with emoji, while we compared two different conditions for brand logos as stimuli. Therefore, we cannot expect to have differences in visual form retrieval. Another reason for this dissimilarity could be the fact that, in addition to visual form, language- or emoji-processing contains extra information such as phonetical, morphological, and lexical, potentially affecting visual form retrieval. Moreover, previous studies found significant theta increase in the temporal lobe with incongruent stimuli. They associate this increase with lexical retrieval^46,52^ or the retrieval of pre-constructed concepts^28^. In our case, it could be that brand logos do not have actual lexical form. Although some brands use words (i.e., dictionary words) or letters in their logo, these elements are not necessarily related lexically to what the brand represents.

This is the first study using time and frequency domain EEG features to investigate how the brain reacts to a mismatch between brand-related cues and the expected brand, represented by its brand logo. In summary, two neuronal markers for semantic violation, the N400 effect and a pronounced theta oscillation, were found. The difference in theta oscillation occurred in the time window of 700–1200 ms, while the ERP difference occurred in the N400 component (i.e., approximately 400 ms). This finding suggests that these two methods capture different aspects of brain activity. Overall, our results were consistent with previous studies investigating semantic violations in other fields. However, specific to our study is the N400 effect present only in the central electrode locations, and pronounced theta in the frontal and central electrode locations. The presence of both markers, associated with the corresponding brain electrode locations, provide strong support for the view that brand logos are not only represented in consumers’ minds but also that this representation differs from other forms of ordinary visual representations (e.g., objects, gestures, emojis). Regarding cognitive processes, we assume a working memory involvement during task performance, because information provided by the brand cues needs to be stored to confront it further with the brand logo information. Though our data did not show theta activation in regions related to long-term memory, as found in previous studies^28^, we infer that this could be because of our stimulus type. We argue that long-term memory must have been present as well. As the task required inferring brand name from brand-related cues, when assessing those cues, participants had to retrieve previous formed associations with those cues from their long-term memory, especially which brand they represented. The same applies for the brand logo. When presented with the logos, it was again necessary to retrieve from memory brand-logo associations. Finally, some form of integration process must have taken place to link those elements (cues, logo, predicted brand name) and reach a decision (the name of the brand). Furthermore, the findings suggest that an error-monitoring process took place during task performance. We presume that the brand cues were in the working memory, together with the brand name information, and when an erroneous (i.e., incongruent) brand logo was shown, the brain engaged in a searching process, trying to find links between the cues and the mismatched brand logo, increasing cognitive load. In summary, our results suggest that brands and their representations (e.g., products and logos) can be deeply encoded in consumers’ minds. Moreover, the data suggests that incongruence between brand cues and brand logos increases consumers’ cognitive load due to the activation of an error-monitoring process.

### Limitations and future studies

In this section, we consider this study’s limitations and discuss suggestions for further research.

i. Our study design required the participants to think about the brand of the products or services that were going to be shown to them. The reminder of this requirement occurred just before the appearance of the target stimuli (and its silent answer) and was intended to reinforce the expectative state, which presupposes a semantic association at the precise moment before the incongruence/congruence appeared. This step was intended to ensure that the participant associated the target with the preceding images. The necessity of this reminder could itself be tested using a control group; this could validate our assumption that the question was implicit at the outset and that a reminder was necessary. In future studies, a comparison could be established between a group that is prompted by the reminder of the “silent question” and a group that is not. Nevertheless, if there were a difference between the two groups, it would remain a challenge to elucidate whether the difference was due to the presence or absence of the reminder question or to other confounding factors (e.g., if the task were unintelligible or lacked a clear goal).
ii. In the incongruent condition, the target brand logo was either a competitor of previously shown brand cues (i.e., from a related product category) or unrelated to the brand cues (i.e., from another product category). In the present study, we did not compare these two conditions due to the limited number of trials of each category. An interesting approach for future research would be to balance these two conditions in a 50–50 proportion in order to investigate this relationship.
iii. Our logos had a linguistic component (i.e., letters/words). To mitigate the possible influence of a reading process in the results, further studies could use either exclusively image-only logos or equally balance the logo types (linguistic and image-only).

## Methodology

### Participants

Thirty-two right-handed participants (13 female) living in Copenhagen of 16 nationalities. Demographics were as follows. Age: *M* 26.84, *SD* 4.33, age range 20–37. Occupation 69% students, 16% workers, 15% both. Highest educational level (completed or ongoing) 12% bachelor, 88% masters. The sample size was determined by a power analysis for the ERP and theta band effect with alpha = 0.05 and power = 80%. The highest sample size required by this analysis was chosen for the study (in this case, N = 32).

All participants signed an informed consent, were debriefed at the end of the experiment, and were paid for their time and effort. The study was approved by the local ethics committee (Technical Faculty of IT and Design, Aalborg University) and performed in accordance with the Danish Code of Conduct for research and the European Code of Conduct for Research Integrity.

### Design and stimuli

A within-subsects design with one independent variable called level of congruence (hereafter, “condition”)—congruent as against incongruent—was conducted. There were 80 image sets in total (40 per condition), where each set was related to a well-known brand. Each image size was 1000 × 1000 px with a white background placed on a black background screen. The presentation order of the sets was randomized across participants. The task was divided into two blocks with 40 image sets each (50% incongruent).

### Data collection and task procedure

Thirty-two channel EEG active electrodes were placed on the scalp of the participant according to the 10–20 system, based on the participant’s head perimeter. The signals were recorded by Brain Products EEG system, using 500 Hz sampling rate. Conductive gel was applied to the electrodes to keep the impedance between the electrodes and the scalp below 25 KΩ (as required by the hardware). A virtual reality (VR) headset (HTC Vive Pro) was placed on top of the EEG cap. The VR headset was used for stimulus presentation because this study was part of a larger study.

**Figure 4:**
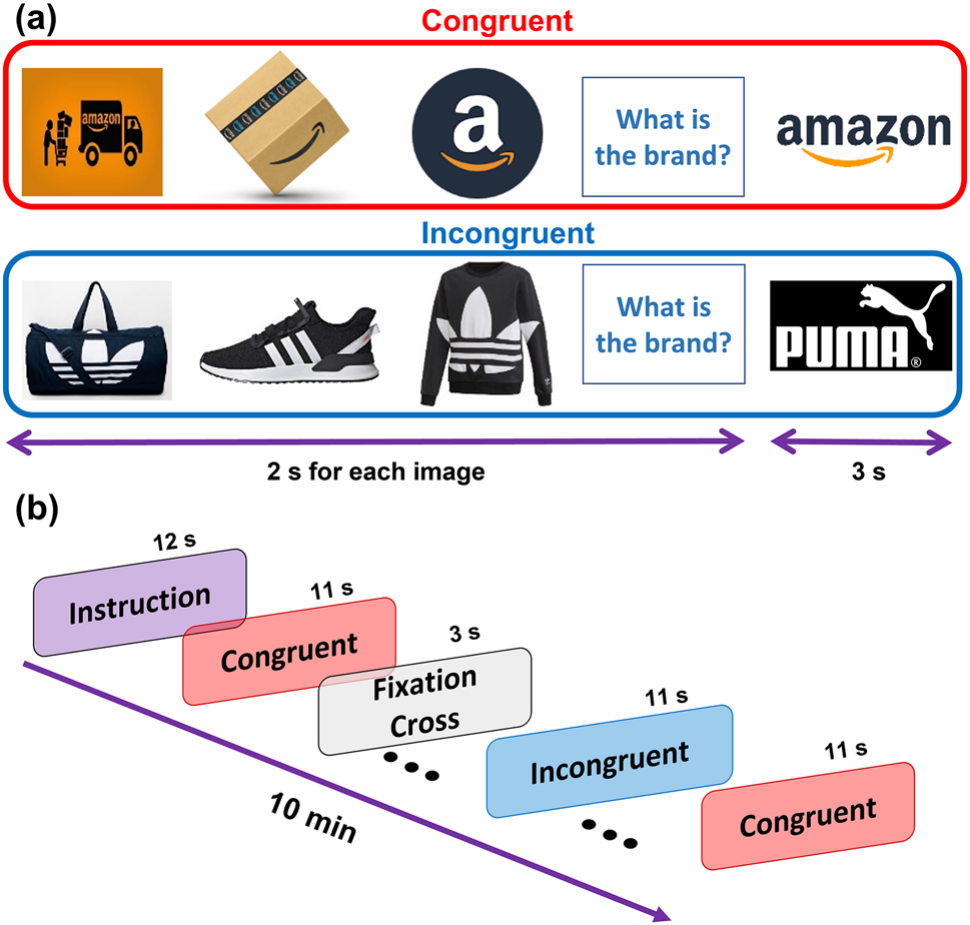
Example for stimuli and the task procedure. Panel (**a**) upper part shows an example for congruent set, where the last image (brand logo) matches the brand of the first three images. Panel (**a**) bottom part is an example for incongruent set where the last image and first three images do not match. Panel (**b**) shows the task procedure, which started with 12 s of instruction followed by congruent and incongruent image sets (randomized through the experiment). After each image set, there was a 3-s fixation cross.

The task comprised 80 image sets (40 incongruent sets). Before the task, participants were informed that they would see a sequence of three images related to a brand and subsequently would be asked to guess the brand. However, they were instructed to think of the answer but not say it aloud. Therefore, each set started by displaying a sequence of three images of a product or service from a specific brand for 2 s each. These three images could include explicit cues (e.g., name of the brand) or only implicit cues (e.g., products or store layout). After the third image, the question “What is the brand?” was displayed for another 2 s. Following this question, the logo was shown for 3 s (this is the target image of our analysis). The logo was either from the brand of the previous images (congruent) or from another brand (incongruent). All logos included a linguistic component (i.e., letters/words) in order to control for possible differences in information processing^39,53^. In the incongruent condition, the mismatched logos were randomized to be either from a competitor brand (20%) or an unrelated brand (80%). Thus, the mismatched logos would violate pre-encoded rules or previous knowledge at the semantic and/or pragmatic levels. Figure 4a shows an example for congruent and incongruent image sets, respectively. Next, a fixation cross appeared for 3 s, and the next image set started. Each block with 40 image sets lasted for almost 10 min (Fig. 4b shows the procedure of each block). The order of congruent and incongruent images was randomized across the participants in each block. The task was presented in the VR environment, using the desktop option of *Steam VR*. The images were therefore seen in 2D, but in a curved, big screen and with the “home” background of the software.

### Data analysis

This section is divided into three sub-sections: (i) pre-processing, (ii) ERP analysis, and (iii) relative power calculation. The analyses were performed using Matlab R2020b (The Math Works, Inc) with in-house codes and tools from EEGLAB 2021.0 (https://eeglab.org/) and FieldTrip 20210128 (http://fieldtriptoolbox.org) toolboxes.

#### Pre-processing

**Figure 5:**
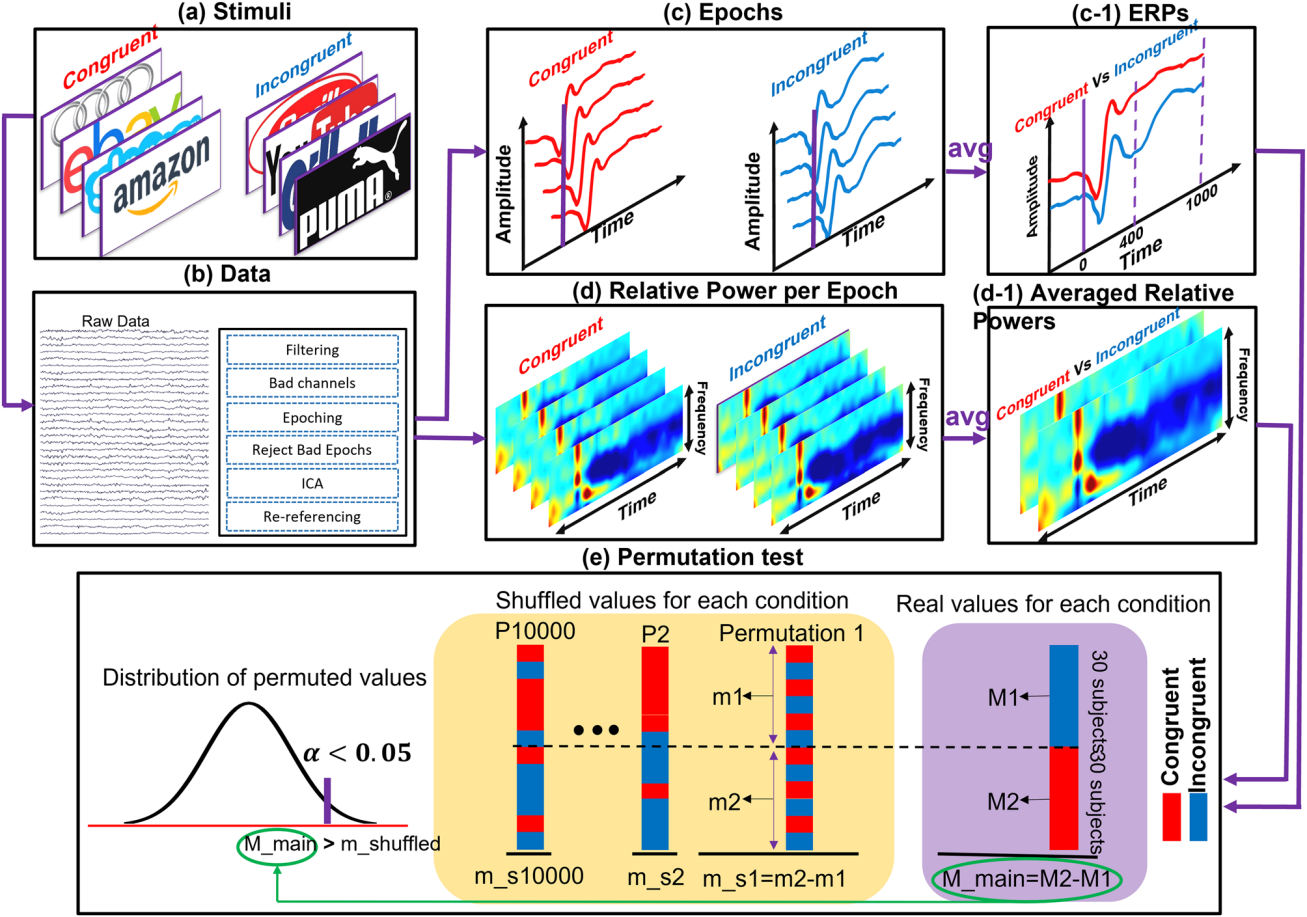
Shows the steps in our methodology. The plots related to congruent condition are indicated by red color, and incongruent condition by blue color. Panel (**a**) shows congruent and incongruent target brands (the last image of each set). Panel (**b**) shows an example of the EEG data followed by pre-processing steps. Panel (**c**) relates to pre-processed epochs corresponding to each condition. By averaging the epochs of each condition we reached panel (**c-1**), showing the ERP activity for each condition. Panel (**d**) shows the time–frequency activity of each epoch for two conditions separately (in each TF, hot colors indicate positive relative power and cold colors indicate negative relative power). By averaging of TF of each condition, panel (**d-1**) is obtained, showing the actual time–frequency activity of each condition. Panel (**e**) shows the statistical analysis using the permutation test—starting with actual difference calculation, then shuffling the extracted feature 10,000 times and calculating the difference in each iteration, and finally building up the random distribution and comparing it with the actual difference.

The signals were filtered using a third order IIR Butterworth filter with 1 to 40 Hz cut-off frequencies to remove high and low frequency noises. Afterward, bad channels were detected using automated rejection procedure with voltage threshold of $\pm$ 500 μV, confirmed by an expert, and rejected from the channel list. All rejected channels were interpolated by spherical spline method using the information from six surrounding channels in FieldTrip toolbox. The average number of rejected channels per participant was 1.43 $\pm$ 1.42. One participant was excluded due to having more than four bad channels. Subsequently, considering the stationary assumption, the filtered data was segmented to 4 s epochs: 1 s before (pre-stimuli) and 3 s after (post stimuli) from the start of the stimulus for each condition (Fig. 5a). Noisy epochs were detected by a strict automatic rejection procedure with a voltage threshold of $\pm$ 120 μV, confirmed by an expert, and rejected from the data. The average number of rejected epochs per subject was 1.46 $\pm$ 2.2. Afterwards, the epochs were concatenated and fed into independent component analysis (ICA) to remove remaining artifacts. The Second-order Blind Identification (SOBI) method was used to estimate source activities. EOG (eye-related artifacts) and other artifact sources were detected by an expert and removed from the source list. For further ERP calculations, the same Butterworth filter, but with low cut-off frequency of 0.1 Hz, was applied to a copy of the raw data, and ERP-filtered data was obtained. The calculated coefficients of ICA part were then applied on the ERP-filtered data to estimate the sources, and the rest of the abovementioned procedure was identical for the ERP analysis. Finally, the de-noised data was re-referenced to the average activity of the electrodes. The preprocessing steps can be seen in Fig. 5b. For the following analyses, we divided the electrodes in four different regions: mid-frontal (Fz, F3, FC1, FC2, and F4), central (C3, CP1, CP2, Cz, and C4), parietal (CP5, CP1, Pz, P3, and P7), and occipital (O1, Oz, and O2).

#### ERP analysis

The ERP-filtered and de-noised data were used to calculate the ERPs. First, the baseline of all epochs was corrected by subtracting, from the entire signal, the signal average across a 200 ms pre-stimulus portion. Then, the epochs of the corresponding conditions (i.e., congruent and incongruent) of specific regions were averaged separately to obtain ERPs per condition and region. The steps are shown in Fig. 5c,c-1. Figure 1a–d shows grand ERP average (obtained from averaging all participants’ ERPs) in different regions. For the purpose of this study, the N400 activity of each individual’s ERP was calculated by averaging from 400 to 600 ms, and these values were then used for statistical analysis.

#### Relative power calculation

The relative power was calculated using the de-noised data. The TF information for each channel was estimated using the Welch method, including a Hanning window with 50% overlap. Then, the baseline-TFs (i.e., the TFs calculated from the one sec pre-stimulus portion of the signal) of all channels from a participant were averaged to obtain the averaged baseline-TF. This obtained average was used to calculate the relative power activity of each epoch as follows:

1 $$relative \; power =\frac{TF -averaged \; baseline \; TF}{averaged \; baseline \; TF}.$$

The calculation steps for relative power are shown in Fig. 5d,d-1. Finally, the relative powers of each condition and each region, for a participant, were obtained by averaging the corresponding TFs separately, and the values were used for statistical analysis. Figure 2 shows the overall TF activity for each condition in the central region.

To select the time window for further statistical analysis, we used a separate permutation test procedure for exploratory searching of power changes; this procedure was an adapted version of a method in the literature^20,54^. To do this, all subjects’ TF in each condition were concatenated separately. Then, the resulting matrices were averaged across subjects. The difference between the two conditions was calculated by subtracting the average TF of the incongruent condition from that of the congruent condition (congruent–incongruent), this is the observed difference. Next, to generate the null distribution, the TFs of the two conditions were scrambled 1000 times, and the difference between the two conditions was calculated for each iteration. Finally, the observed difference was compared to the generated null distribution in order to calculate the p-value for each pixel of TF difference^55^. Then, the pixels that had a p-value lower than 0.01 were considered to show a significant difference between the two conditions. The result of this procedure in the central and mid-frontal region revealed that TF difference showed a significant pattern within the 700–1200 ms time window. Therefore, this interval was used for further statistical analysis.

#### Statistical analysis

The dependent variables are the N400 feature from ERP and the TFs, which are the averaged TF for each individual in the theta band (4 to 7 Hz) and from 700 to 1200 ms. We used a permutation test to evaluate the significant differences between the conditions. As shown in Fig. 5e, the actual difference (M_main) between extracted features for each condition was calculated. Then, the features corresponding to each condition were shuffled 10,000 times, and in each shuffled trial, the difference (m_s1 to m_s10000) between two newly generated groups was calculated. These differences were used to generate a random distribution, and the actual difference was tested on this distribution using significance level of 0.05.

## References

[1] Statista Research Department. Brand value—statistics & facts. *Advertising & Marketing, Brands & Leaders* https://www.statista.com/topics/1664/brand-value/ (2021). Accessed 14 Sep 2021.

[2] Cobb-Walgren CJ, Ruble CA, Donthu N (1995). Brand equity, brand preference, and purchase intent. J. Advert..

[3] Laroche M, Kim C, Zhou L (1996). Brand familiarity and confidence as determinants of purchase intention: An empirical test in a multiple brand context. J. Bus. Res..

[4] McClure SM (2004). Neural correlates of behavioral preference for culturally familiar drinks. Neuron.

[5] Reimann M, Castaño R, Zaichkowsky J, Bechara A (2012). How we relate to brands: Psychological and neurophysiological insights into consumer–brand relationships. J. Consum. Psychol..

[6] Posner, R. Syntactics, semantics, and pragmatics revisited half a century after their introduction by Charles W. Morris. In *Signs of Humanity/L’homme et ses signes* (eds. Deledalle, G., Balat, M. & Deledalle-Rhodes, J.) 1349–1354 (De Gruyter Mouton, 1992). 10.1515/9783110854572-168.

[7] Van Berkum, J. J. A. The neuropragmatics of ‘simple’ utterance comprehension: An ERP review. In *Semantics and Pragmatics: From Experiment to Theory* (eds. Sauerland, U. & Yatsushiro, K.) 276–316 (Palgrave Macmillan, 2009).

[8] Baggio G, Hagoort P (2011). The balance between memory and unification in semantics: A dynamic account of the N400. Lang. Cogn. Process..

[9] Kutas M, Hillyard SA (1980). Reading senseless sentences: Brain potentials reflect semantic incongruity. Science (80-)..

[10] Hanslmayr S (2008). The electrophysiological dynamics of interference during the stroop task. J. Cogn. Neurosci..

[11] Tang D, Hu L, Li H, Zhang Q, Chen A (2013). The neural dynamics of conflict adaptation within a look-to-do transition. PLoS One.

[12] Ergen M (2014). Time–frequency analysis of the event-related potentials associated with the Stroop test. Int. J. Psychophysiol..

[13] Appelbaum LG, Boehler CN, Davis LA, Won RJ, Woldorff MG (2014). The dynamics of proactive and reactive cognitive control processes in the human brain. J. Cogn. Neurosci..

[14] Shitova N, Roelofs A, Schriefers H, Bastiaansen M, Schoffelen J-M (2016). Using brain potentials to functionally localise Stroop-like effects in colour and picture naming: Perceptual encoding versus word planning. PLoS One.

[15] McKay CC, van den Berg B, Woldorff MG (2017). Neural cascade of conflict processing: Not just time-on-task. Neuropsychologia.

[16] Zhang Q, Lawson A, Guo C, Jiang Y (2006). Electrophysiological correlates of visual affective priming. Brain Res. Bull..

[17] Chen X, Yuan J, Guo J, You Y (2013). Neural oscillatory evidence of the difference between emotional and conceptual processing in language comprehension. Neurosci. Lett..

[18] Wu YC, Coulson S (2005). Meaningful gestures: Electrophysiological indices of iconic gesture comprehension. Psychophysiology.

[19] Ousterhout, T. N400 congruency effects from emblematic gesture probes following sentence primes. In *2015 IEEE 19th International Conference on Intelligent Engineering Systems (INES)* 411–415 (IEEE, 2015). 10.1109/INES.2015.7329744.

[20] Hald LA, Bastiaansen MCM, Hagoort P (2006). EEG theta and gamma responses to semantic violations in online sentence processing. Brain Lang..

[21] Wang L, Zhu Z, Bastiaansen M (2012). Integration or predictability? A further specification of the functional role of gamma oscillations in language comprehension. Front. Psychol..

[22] Ghosh Hajra S (2018). Multimodal characterization of the semantic N400 response within a rapid evaluation brain vital sign framework. J. Transl. Med..

[23] Mongelli V, Meijs EL, van Gaal S, Hagoort P (2019). No language unification without neural feedback: How awareness affects sentence processing. Neuroimage.

[24] Weimer NR, Clark SL, Freitas AL (2019). Distinct neural responses to social and semantic violations: An N400 study. Int. J. Psychophysiol..

[25] Kutas M, Hillyard SA (1984). Brain potentials during reading reflect word expectancy and semantic association. Nature.

[26] Bentin S, Kutas M, Hillyard SA (1993). Electrophysiological evidence for task effects on semantic priming in auditory word processing. Psychophysiology.

[27] Coco MI, Araujo S, Petersson KM (2017). Disentangling stimulus plausibility and contextual congruency: Electro-physiological evidence for differential cognitive dynamics. Neuropsychologia.

[28] Tang M, Zhao X, Chen B, Zhao L (2021). EEG theta responses induced by emoji semantic violations. Sci. Rep..

[29] Barrett SE, Rugg MD (1990). Event-related potentials and the semantic matching of pictures. Brain Cogn..

[30] Kutas M, Federmeier KD (2011). Thirty years and counting: Finding meaning in the N400 component of the event-related brain potential (ERP). Annu. Rev. Psychol..

[31] de Chernatony L, Dall’Olmo Riley F (1998). Defining A ‘Brand’: Beyond the literature with experts’ interpretations. J. Mark. Manag..

[32] Van Horen F, Pieters R (2012). When high-similarity copycats lose and moderate-similarity copycats gain: The impact of comparative evaluation. J. Mark. Res..

[33] Hamm JP, Johnson BW, Kirk IJ (2002). Comparison of the N300 and N400 ERPs to picture stimuli in congruent and incongruent contexts. Clin. Neurophysiol..

[34] Kutas M, Federmeier KD (2000). Electrophysiology reveals semantic memory use in language comprehension. Trends Cogn. Sci..

[35] Beatty PJ, Buzzell GA, Roberts DM, McDonald CG (2020). Contrasting time and frequency domains: ERN and induced theta oscillations differentially predict post-error behavior. Cogn. Affect. Behav. Neurosci..

[36] Brunetti M, Zappasodi F, Croce P, Di Matteo R (2019). Parsing the Flanker task to reveal behavioral and oscillatory correlates of unattended conflict interference. Sci. Rep..

[37] Herweg NA, Solomon EA, Kahana MJ (2020). Theta oscillations in human memory. Trends Cogn. Sci..

[38] Luo Y, Zhang Y, Feng X, Zhou X (2010). Electroencephalogram oscillations differentiate semantic and prosodic processes during sentence reading. Neuroscience.

[39] Morgan C, Fajardo TM, Townsend C (2021). Show it or say it: How brand familiarity influences the effectiveness of image-based versus text-based logos. J. Acad. Mark. Sci..

[40] Kutas, M. & Van Petten, C. In *Advances in Psychophysiology* (eds. Ackles, P.K., Jennings, J.R., & Coles, M.G.H.) 139–187. (1988).

[41] Sitnikova T, Kuperberg G, Holcomb PJ (2003). Semantic integration in videos of real-world events: An electrophysiological investigation. Psychophysiology.

[42] McPherson WB, Holcomb PJ (1999). An electrophysiological investigation of semantic priming with pictures of real objects. Psychophysiology.

[43] West WC, Holcomb PJ (2002). Event-related potentials during discourse-level semantic integration of complex pictures. Cogn. Brain Res..

[44] Davidson DJ, Indefrey P (2007). An inverse relation between event-related and time-frequency violation responses in sentence processing. Brain Res..

[45] Hagoort P, Hald L, Bastiaansen M, Petersson KM (2004). Integration of word meaning and world knowledge in language comprehension. Science (80-)..

[46] Bastiaansen MCM, Van Der Linden M, Ter Keurs M, Dijkstra T, Hagoort P (2005). Theta responses are involved in lexical—Semantic retrieval during language processing. J. Cogn. Neurosci..

[47] Zion-Golumbic E, Kutas M, Bentin S (2010). Neural dynamics associated with semantic and episodic memory for faces: Evidence from multiple frequency bands. J. Cogn. Neurosci..

[48] Summerfield C, Mangels JA (2005). Coherent theta-band EEG activity predicts item-context binding during encoding. Neuroimage.

[49] Morís Fernández L, Torralba M, Soto-Faraco S (2018). Theta oscillations reflect conflict processing in the perception of the McGurk illusion. Eur. J. Neurosci..

[50] Pan F, Ou Y, Sun H, Qian Y (2020). Integration of conflict resolution and positive emotions: Electrophysiological evidence. Neuropsychologia.

[51] Ma J, Liu C, Chen X (2015). Emotional conflict processing induce boosted theta oscillation. Neurosci. Lett..

[52] Klimesch W (1999). EEG alpha and theta oscillations reflect cognitive and memory performance: A review and analysis. Brain Res. Rev..

[53] Ehri LC (2005). Learning to read words: Theory, findings, and issues. Sci. Stud. Read..

[54] Maris E (2004). Randomization tests for ERP topographies and whole spatiotemporal data matrices. Psychophysiology.

[55] Song H, Finn ES, Rosenberg MD (2021). Neural signatures of attentional engagement during narratives and its consequences for event memory. Proc. Natl. Acad. Sci..

